# Economics of Twin Pregnancies in Dairy Cattle

**DOI:** 10.3390/ani11020552

**Published:** 2021-02-20

**Authors:** Victor E. Cabrera, Paul M. Fricke

**Affiliations:** Department of Animal and Dairy Sciences, University of Wisconsin, Madison, WI 53706, USA

**Keywords:** twinning, dairy cows, economics

## Abstract

**Simple Summary:**

Twinning in dairy cattle is not desirable due to negative effects on both cows that calve twins and calves born as twins that result in economic losses to dairy farms. Although a twin pregnancy could bring additional income from extra calves and it could shorten gestation length, twinning compromises milk production, increases the incidence of dystocia and perinatal mortality, decreases calf birth weight, increases the incidence of metabolic diseases, decreases fertility, increases the incidence of freemartinism, increases overall culling risks, and shortens the productive lifespan of cows. Based on a summary of economic analyses from several studies, the estimated losses due to twinning range between $59 to $161 per twin pregnancy. When twins are diagnosed early during gestation, management options might include doing nothing, terminating the pregnancy, or attempting manual embryo reduction. Based on a recent economic analysis of these options, attempting manual embryo reduction decreased the economic losses of a twin pregnancy by $23 to $45.

**Abstract:**

Twinning in Holstein dairy cows has increased over time concurrent with increased milk production. Twinning in dairy cattle is not desirable due to the negative effects on both cows that calve twins and calves born as twins that result in economic losses to dairy farms. Although a twin pregnancy could bring additional income from extra calves and shorten gestation length, twinning compromises milk production, increases the incidence of dystocia and perinatal mortality, decreases calf birth weight, increases the incidence of metabolic diseases, decreases fertility, increases the incidence of freemartinism, increases overall culling risks, and shortens the productive lifespan of cows. Based on a summary of economic analyses from several studies, the estimated losses due to twinning range between $59 to $161 per twin pregnancy. Most twinning in dairy cows is dizygotic and directly related to the incidence of double ovulations, and economic losses are greater for unilateral than for bilateral twins. Hormonal manipulation before artificial insemination that allows for timed artificial insemination is a primary strategy for decreasing twinning in dairy cows before it occurs by decreasing the incidence of double ovulation thereby decreasing conception of dizygotic twins and the associated negative economic consequences. When twins are diagnosed early during gestation, management options might include doing nothing, terminating the pregnancy, or attempting manual embryo reduction. Based on a recent economic analysis of these options, attempting manual embryo reduction decreased the economic losses of a twin pregnancy by $23 to $45.

## 1. Introduction

**Figure 1:**
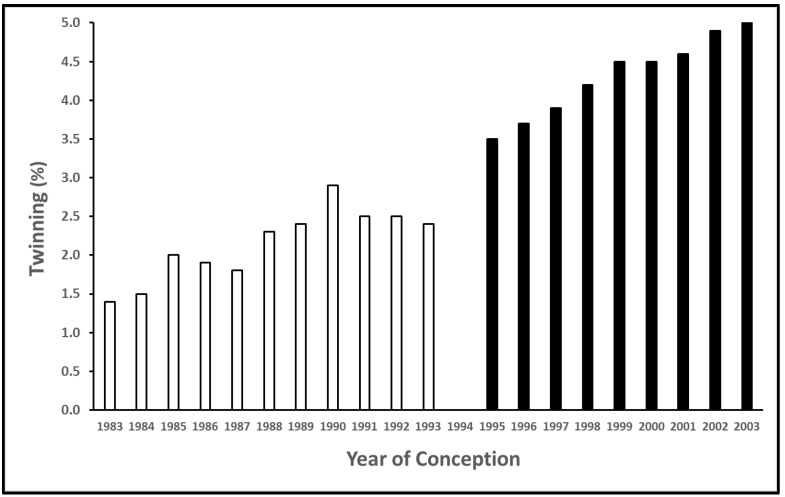
Trend in reported twinning rate in Holstein cows in the USA. from 1983 to 2003. Data includes nonlactating and lactating Holsteins. Open bars: data adapted from [6]; Solid bars: data adapted from [7].

Cattle are a monotocous species, which means that, under most circumstances, a successful pregnancy results in the birth of one calf. However, occasionally, the reproductive process in cattle results in the birth of twins. Under some beef cattle production scenarios, twinning might be considered a desirable trait that enhances profitability by increasing weaned calf weight that is produced per cow [1,2]. By contrast, twinning in dairy cattle is an undesirable trait that reduces the overall profitability of a dairy operation through negative effects on cows calving twins as well as on calves born as twins. The risk factors for twinning include genetics, season, parity, ovulation rate, and milk production [3]. More recently, low concentrations of progesterone during selection of the dominant preovulatory follicle has been associated with an increase in the incidence of double ovulations resulting in dizygous twins [4,5]. Based on an epidemiologic study of twinning [6], twinning in the USA. increased over a 10-year period from 1983 to 1993. The authors implicated the concurrent increase in milk production during the study period as the single most important factor that is associated with the increase in twinning [6]. We published an observational analysis of twin births in Holstein cows in the Upper Midwest region of the USA from 1996 to 2004 to determine whether this trend for an increase in twinning has continued over time [7]. A data set of Holstein calving records from January 1996 to September 2004 comprising 4103 herds with 2,304,278 calving events representing 1,164,233 cows and 96,069 twin births was extracted from Minnesota DHIA archives to assess the reported twinning trends across time. Overall, the reported twinning rate averaged 4.2%, and twinning increased with parity (1.2% for nulliparous vs. 5.8% for multiparous cows) and with time (3.4% in 1996 to 4.8% in 2004). Figure 1 shows the trend for increased twinning across time in the USA. Holstein population from 1983 to 1993 based on the data from [6] and from 1996 to 2003 based on data from [7]. Clearly, twinning in Holsteins has consistently increased from 1983 to 2003 (Figure 1). Based on this long-term trend, we may expect twinning in Holsteins to continue to increase over time if genetic selection for higher milk production continues. 

The consequences of twinning are severe for both the cow calving twins and calves born as twins. Twinning decreases reproductive performance by increasing average days open and services per conception during the subsequent lactation [8]. Cows calving twins are at greater risk for many reproductive disorders, including retained placenta, dystocia, and metritis [8,9,10,11], as well as metabolic disorders, including displaced abomasum, and ketosis [8,11,12]. Not surprisingly, cows calving twins are culled at a greater rate than non-twinning herd mates [13,14]. The incidence of abortion, stillbirth, neonatal calf mortality, and reduced birth weight are greater among twin than singleton calves, probably due to decreased gestation length and increased incidence of dystocia among cows calving twins [8,9,12,15,16,17]. The focus of this review is on the economic impact of twinning on dairy farms, an overview of potential management strategies to either avoid or mitigate the negative impact of twinning, and to summarize a recent economic analysis that compared the outcomes of these management interventions for cows that are diagnosed with twin pregnancies. 

## 2. Economic Impacts of Twinning

One of the first economic studies that was published in the early 90’s reported that twin pregnancies in dairy cattle resulted in an average loss of income of $112/cow per lactation (1£ = 1.33US$) or 15% deficit as compared to their herdmates calving singletons ([13]; Table 1). This aggregated loss value was the result of a greater incidence of retained placenta, longer calving intervals, and much greater culling risks of twin bearing cows than their herdmates, even though they produced more milk [13]. Before [13], studies on the impact of twinning were limited, did not report economics, and were not fully conclusive, although they did point out that cows with twin pregnancies had shorter gestations, which led to more stillbirths, an increased risk of retained placenta, and some level of increased milk production [18,19]. A few months after [13], ref. [20] reported a loss of $108 per twin birth as the difference of $171 in total losses, which were partially offset by additional calf revenues of $63 (Table 1). They performed a sensitivity analysis and concluded that, in no realistic situation, it would be economically beneficial to promote twin pregnancies in dairy cows. In contrast to [13], ref. [20] reported a negative milk production after twining, which was included as a negative value in the partial budgeting analysis. 

After decades of little research on the economic impact of twinning, ref. [21] presented a review study, in which they concluded that twinning is a double-edged sword, because it presents some pros and many cons that cannot be easily reconciled. Economically negative factors included increased incidence of abortions, ketosis, mastitis, dystocia, lower birth weight, and stillbirths; lower fertility; increased risk of culling; and, reduced milk yield. Cows calving twins had 0.78 pregnancy and 1.42 culling chances as compared to cows calving singletons. They concluded that it is not profitable to promote or select for twin pregnancies in dairy cattle and projected that the cumulative losses due twin pregnancies in US dairy herds was $55 million annually while assuming 5% incidence of twinning and $110 in economic losses per twin pregnancy. 

The advent of transrectal ultrasonography for dairy cattle reproduction introduced a new tool to more accurately assess and classify twinning in dairy cows [22]. The presence of two or more CL on the ovaries at the time of an early pregnancy diagnosis can be used to identify cows carrying twins [22] because of the advances in the resolution of ultrasound scanners and because ~95% of twins in dairy cows are dizygotic [23]. The identification of twins early during gestation based on the presence of multiple CL and location of the embryos within the uterus can be conducted using ultrasound [22]. Thus, dizygous twin pregnancies can be classified based on laterality as either unilateral (both twins in the same uterine horn), or bilateral (twins in opposite uterine horns). Based on this knowledge, ref. [24] conducted a more complete economic analyses of twinning, studying not only the economic impact of type of twinning (unilateral vs. bilateral), but also the value of potential management strategies to mitigate the negative effects of twinning, including pregnancy termination or manual embryo reduction. They calculated the economic negative impact of twinning to be on average $161 with a range between $97 and $225, depending on type of twinning, parity, and DIM (Table 1). They estimated that twinning on USA. dairy farms had a negative impact of $96 million/year and yet reported that the best option to deal with a twin pregnancy after diagnosis was to attempt manual embryo reduction, as described by [25]. 

Another study, the most recent we found in the literature, ref. [26] also assessed economic impacts of twin pregnancies in large Hungarian herds and concluded that the overall loss due to a twin pregnancy added $59 per twin pregnancy (1EU = 1.18US$; Table 1). This loss was the result of increased calving to conception interval, increased service per conception, reduced conception rate, and increased incidence of retained placenta. 

## 3. Economic Models to Assess Twining

Ref [13] used partial budgeting as the methodological framework to compare the performance of 403 cows that calved twins with cohort cows that calved singletons. Cohorts were defined within the same farm, having the same parity, and calving within the same 14-day period. From these 403 cohort groups, complete datasets were not available for all of the factors studied, so they used the available data in each factor to evaluate significant differences in the following economic-impacting factors: before or during calving: (1) gestation length, (2) calving problems, (3) stillbirths, (4) culling risk; and after calving (5) fertility, and (6) milk yield. From these, milk yield, which was numerically greater for cows calving twins, but not significantly different from cows calving singletons, was included in the partial budgeting: numerical differences were multiplied by a marginal income over feed cost value in favor of the twin pregnancies. By contrast and in spite of the fact that they reported 5 percentage points more abortions (significant difference) for twin than for singleton pregnancies, they did not include its possible negative economic impact. Other factors, including days open, increased services per conception, or increased culling because of reproductive failure, were assumed to capture these costs. The losses were then calculated for the subsequent lactation, including 25 extra days to calving, 0.5 more services per conception, 14% greater risk of culling, and veterinary services for extra 14% retained placenta and 20% vulval discharge. These losses were partially decreased by 0.75 extra calves sold and 235 L extra milk produced. The losses were estimated at $212, while revenues were estimated at $100 to equal a net loss of $112 per twin pregnancy per cow per lactation (Table 1). This figure would be $150 per cow per lactation if it would exclude the potential gains for additional milk yield, which did not differ significantly. They alluded to yet another potential negative cause that they were not able to study, the lower body weight and condition of twin calves and the suspicion that cow twin bearers would calve to a very low body condition. They concluded that induced twinning should not be considered to be an option for dairy cattle. 

Ref [20] developed a model based on data from 381 twin calvings [8], in which did not find significant differences or did not use increased metabolic diseases for twinning as a negative effect after calving. In their economic estimates, the only positive factor for twinning was the additional production of calves, which were penalized for being, on average, 10 kg lighter than calves that were born as singletons and to have 14 percentage points more stillbirth mortality rate (19% vs. 5%). An important loss included was decreased milk production (as opposed to [13]). Although the data indicated that cows calving twins produced 100 kg more milk during the first 100 DIM of the lactation in which they were pregnant with twins, they assumed that these cows were already higher producers, so the additional production was considered not to be an effect of the twinning. This would agree with studies in which the incidence of double ovulations dramatically increases as milk production near the time of ovulation increases [27,28]. However, milk compromised the rest of the lactation and, during the following lactation, which were assessed as the marginal value (milk price minus feed cost) as a loss revenue for twinning cows. Other costs of twinning were: (1) abortion, which was calculated as twinning pregnancies having 0.8 calvings of singleton calvings; (2) fertility treatment that included the cost of hormonal treatments pre breeding, treatments for diseases in greater incidence by twinning, such as retained placenta or endometritis; (3) enlarged calving interval; and, (4) premature culling according to parity and lactation stage.

Ref [20] reported $63 gains from extra calves, but $171 losses when cows calved twins as compared to cows that calved singletons (Table 1). Therefore, the estimated net loss was $108 per twin calving. The negative economic factors were milk reduction ($101, 59%), premature culling ($40, 23%), and fertility issues, including enlarged calving interval ($31, 18%). The loss from milk minus feed cost was estimated at $26 during the twinning pregnancy and $75 in the lactation after. Additionally, calving interval loss was the difference of shorter gestation during twin pregnancy minus more days open in subsequent lactation ($4 − $10 = −$6). Premature culling was heavily concentrated in later lactations and either earlier or later lactation stages. The cost of abortion was composed of missing calves ($13) and additional milk reduction ($6). Single factor sensitivity analysis demonstrated that, within plausible and realistic ranges, no breakeven of twinning vs. singleton pregnancy was possible, which highlighted the fact that twining is an undesirable trait in dairy cattle. Two important points were recommended as continued research. The first one was the assumptions of milk depression during the actual gestation, which was in the opposite direction, as expected (first 100 DIM), and at the end was not significantly different. The authors attributed it to the lack of data records and the actual demographics of the cows. The losses would still be large enough for their conclusions to hold, even if removing the possible loss of income of the twin gestation milk. The second recommendation was that the weight of the calves was an important factor for which they used literature assumptions. They recommended recording calves’ weights for better assessments.

Ref [26] analyzed data from 3,660 calvings from five large dairy farms in Hungary through linear and logistic regression and Dunnett test. For the economic analysis, they included losses due to extra days open and excess semen use. They found that the calving interval increased by 12.8 days and service per conception by 2.8 times per case of twin pregnancy, which added to a loss of $59 per case (Table 1).

Ref [21] reviewed the literature regarding the possible causes and consequences of twin calving during the gestation and in the subsequent lactation. They reported that twin pregnancies have a shorter gestation period, better reproductive performance, and shorter calving interval (desirable traits). However, cows calving twins suffer greater dystocia and perinatal mortality, lower fertility in subsequent lactation, a greater incidence of freemartinism, greater culling risks, and, therefore, shorter lifespan, all of which have important negative economic implications. Regarding milk yield, which is probably the most important factor in determining profit of management strategies, they reported that reports in the literature are equivocal. More recent reports indicate that milk yield decreases after twin-birth calving [29], although there are indications of the opposite [13,30].

## 4. Management Strategies to Avoid Twinning

Dairy farmers have widely adopted hormonal synchronization protocols, such as Ovsynch, for use in reproductive management programs for dairy cows [31], and the use of synchronization protocols for inseminating dairy cows in the USA. has increased over time [32]. After over 20 years of research, a deeper understanding of the physiology underlying the original Ovsynch protocol has allowed for a dramatic increase in fertility to timed AI in high producing Holstein cows, resulting in the concept of fertility programs, such as the Double-Ovsynch protocol [33]. Contrary to the idea that reproductive hormones increase twinning, hormonal manipulation of cows, so that progesterone was increased during development of the ovulatory follicle resulted in a decreased incidence of double ovulation [34,35]. More recently, two studies manipulated circulating progesterone concentrations in high-producing Holstein cows, so that the preovulatory follicular wave is initiated in either a low or a high progesterone environment. In the first study [4], the incidence of double ovulation was three-fold greater for low than for high progesterone cows (33% vs. 10%), which resulted in more twin pregnancies 32 d after TAI for Low-P4 than for High-P4 cows (29% vs. 0%) and less pregnancy loss. In a second study [5], cows that were manipulated into a low progesterone environment during growth of the ovulatory follicle had a 49% incidence of double ovulation, P/AI of 66.4%, and pregnancy loss from day 23 to calving of 33%. Pregnancy losses during early gestation are three-fold greater for cows conceiving twins than for cows conceiving singletons [15,36]. Taken together, twinning in high producing Holstein cows may be a major cause of pregnancy losses being observed on dairy farms [5]. Interestingly, ~10% of cows in the high progesterone treatments in both experiments had double ovulations, suggesting that factors other than progesterone, such as genetics or monozygotic twinning, may likely occur. Overall, increasing progesterone during growth of a synchronized preovulatory follicular wave dramatically decreased the double ovulation rate while increasing fertility and decreasing subsequent pregnancy loss. By contrast, a study using progesterone manipulating protocols that are similar to those reported by [4], but conducted in lower producing Irish Friesian Holsteins, did not result in increases in double ovulation or twinning, further implicating the role of high milk production in modern dairy cows in twinning [37]. 

Because milk production is highly correlated (r = 0.88) with feed intake in high producing dairy cows [38], increased hepatic blood flow resulting from high feed intake provides a physiological mechanism for decreased circulating P4 concentrations in lactating dairy cows through hepatic metabolism of steroids [39]. The Double-Ovsynch protocol [40,41] effectively presynchronizes cows and manipulated ovarian function to maximize progesterone concentrations during growth of the preovulatory follicle [42] and decrease the double ovulation rate and subsequent dizygotic twinning rate in high-producing Holsteins. We conducted an economic evaluation among seven reproductive programs and including a sensitivity analysis of the cost of hormonal treatments [43]. In the first analysis, we calculated the economic impact of switching from a Presynch-Ovsynch program to a Double-Ovsynch program (Double-Ovsynch+PGF). The Double-Ovsynch+PGF program was more profitable than other compared programs, including a Presynch–Ovsynch program with 100% timed AI or a Presynch–Ovsynch program that incorporated detection of estrus, despite the higher upfront cost incurred by using more hormonal treatments. In a second analysis, we conducted a break-even analysis in which the cost of hormonal treatments was incrementally increased within various reproductive management programs. The advantage for the Double-Ovsynch+PGF program remained until the cost of hormones was increased five to 14 times current USA. market prices and two to six times current European market prices. We concluded that more intensive reproductive programs that use more hormonal treatments, but result in substantially increased reproductive performance, are more profitable than less intensive programs and remain so, even if hormone prices are unusually high [43]. Thus, hormonal manipulation that allows for timed artificial insemination may be a primary strategy to decrease twinning in high producing dairy cows before it occurs by decreasing the incidence of double ovulation, thereby decreasing the conception of dizygotic twins and avoiding the associated negative economic consequences of twinning. 

## 5. Management Strategies to Mitigate Negative Effects of Twinning

Despite the use of hormonal strategies, such as Double-Ovsynch to decrease the incidence of double ovulations and dizygotic twinning at first insemination, some twinning in dairy herds will persist due to other factors, including genetics and monozygotic twinning. Cows that are diagnosed with twins on farms using transrectal ultrasonography present a conundrum for both farmers and veterinarians due to the negative consequences of deciding to do nothing. Several management interventions that have been considered include pregnancy termination, selective reduction, and nutritional management during the transition period. 

### 5.1. Pregnancy Termination

One method for dramatically reducing or eliminating twinning in a dairy herd is to identify cows carrying twins and induce pregnancy loss by the administration of a luteolytic agent such as prostaglandin F_2α_. For singleton pregnancies, treatment with a luteolytic dose of prostaglandin F2_α_ at 39 d in gestation decreased progesterone within 24 h and caused an expulsion of the conceptus in all cows within 48 h [44]. However, there are several arguments against proactive termination of all twin pregnancies identified early in gestation. First, the economic loss incurred due to pregnancy loss has been estimated to range from $46 [45] to $300 [46,47]. Because the incidence of twinning increases with increasing milk production, cows that are diagnosed with twins are often the highest producing cows in the herd that incur the greatest economic loss associated with pregnancy loss. Second, although estimates for heritability and repeatability for twinning in dairy cows are low (0.08 and 0.09, respectively; [48,49], a prior incidence of twinning is a risk factor for subsequent twin births [8,50]. Third, pregnancy loss before 90 d in gestation for cows with unilateral twins did not differ between the control cows and cows that wee subjected to manual amnion rupture followed by progesterone treatment for 21 d [25], whereas pregnancy loss for cows carrying bilateral twins [36] was similar to that reported for Holsteins overall [51]. Finally, bilateral dizygotic twins had increased survival and body weight at birth, a longer gestation length, and less dystocia than unilateral dizygotic twins [52]. Based on these data, a possible strategy would be to allow cows that are diagnosed with bilateral twins to continue gestation, whereas selective reduction could be attempted for cows that were identified with unilateral twins. Because twin and triplet births had a greater incidence of dystocia than single births [52], cows that are diagnosed pregnant with bilateral twins should be provided extra assistance at calving.

### 5.2. Selective Reduction

Selective embryo reduction early during gestation has been used to mitigate the potentially dangerous maternal effects of multiple births in both women [53] and mares [54]. Reasonable success has been reported in mares when one twin was manually crushed when the procedure was performed before 30 d in gestation [55]. Two controlled experiments reported the efficacy of using manual crushing of the amnion of one of the embryos to maintain a viable singleton pregnancy [25,36]. In the first experiment [36], 33 cows that were identified with unilateral twins were randomly assigned to one of three treatments: (1) untreated controls; (2) manual amnion rupture; and, (3) manual amnion rupture plus progesterone treatment (PRID containing 1.55 g progesterone) for 28 d. Embryo reduction was attempted between 35 to 40 d in gestation, because most cows undergo a spontaneous reduction of twins at this time [36]. Pregnancy loss for control cows (i.e., spontaneous loss of both twins) was 27% (3/11), whereas pregnancy loss for cows undergoing manual amnion rupture was 100% (11/11). Interestingly, pregnancy loss for cows that were treated with progesterone for 28 d after amnion rupture was 55% (6/11). One embryo from a cow treated with progesterone after manual amnion rupture survived and the cow calved twins, whereas the remaining five cows calved singletons [36]. A follow-up experiment was conducted to evaluate the effect on pregnancy maintenance of embryo reduction via manual rupture of the amnion in Holstein cows that were diagnosed with both unilateral and bilateral twin pregnancies ([25]; Table 2). At 35 to 41 d of gestation, 55 cows identified with live twins using transrectal ultrasonography were blocked by laterality, and randomly assigned to manual reduction followed by treatment with progesterone (PRID containing 1.55 g progesterone) for 21 d or served as untreated controls, in which no manipulation was done. Pregnancy loss before 90 d in gestation did not differ between treatments and occurred in 32% of control cows and 41% of cows after manual amnion rupture followed by progesterone for 21 d. Independent of treatment, the risk of pregnancy loss was 8.7 times greater for unilateral as compared to bilateral twin pregnancies, yet pregnancy loss did not differ between control cows with unilateral twins and unilateral twin reduction cows (62% vs 54%, respectively). By contrast, 29% of cows with bilateral twin pregnancies that were subjected to twin reduction lost their pregnancies, whereas no losses occurred in control cows with bilateral twin pregnancies. Overall, 44% (12/27) of cows subjected to manual amnion rupture went on to calve singletons as compared to 54% (15/28) of control cows that went on to calve twins. The authors concluded that embryo reduction by manual amnion rupture, followed by progesterone treatment, did not experience an additional risk of pregnancy loss for unilateral twin pregnancies, whereas it increased the risk of pregnancy loss in bilateral twin pregnancies [25]. 

### 5.3. Nutritional Management during the Transition Period 

Energy demands during gestation are 50% to 70% greater for cows carrying twins when compared to singletons [56,57], yet cows carrying twins have less prepartum DMI than herd mates carrying singletons [58]. In addition, cows carrying twins have a decreased gestation length and therefore are less likely to experience a full three-week exposure to a close-up diet during the dry period [8]. Thus, the feeding management strategies may offer an opportunity to mitigate the negative effects of twinning in dairy cattle [59]. An experiment was conducted in order to evaluate the effect of dry period feeding management on metabolic status and lactation performance in Holstein cows carrying singleton vs. twin pregnancies [60]. Dry period feeding management consisted of feeding a moderate-energy close up diet throughout the entire dry period (eight-week close-up) versus feeding a far-off diet from 60 to 21 d before expected calving date followed by a close-up diet until calving (three-week close-up). The treatments were arranged in a 2 × 2 factorial design with a randomized block design that included 47 Holstein cows. Our hypothesis was that increasing the duration of feeding a close-up diet during the dry period would improve the metabolic status and lactation performance for cows carrying twins, but not for cows carrying singletons. Contrary to the hypothesis, the metabolic response to dry period feeding strategy was independent of twin status [60], indicating that altering nutritional management to increase energy during the dry period did not improve metabolic status for cows carrying twins. Interestingly, there was an effect of diet, in which cows that were fed a moderate energy diet throughout the entire dry period had greater milk production as compared to cows fed according to the NRC (2001) energy requirements for the entire dry period (i.e., a far-off diet followed by a close-up diet for three weeks). Based on these results [60], differential management of cows carrying twins during the dry period did not improve the metabolic status of cows carrying twins.

## 6. Economics of Twinning Management Strategies

Based upon the concepts for either terminating twin pregnancies or attempting manual embryo reduction, ref. [24] developed a probabilistic decision tree model to calculate the economic value of a singleton pregnancy and three interventions for a twin pregnancy: (1) do-nothing, (2) terminate the pregnancy, or (3) attempt manual embryo reduction. They followed a virtual farm for 1,400 consecutive days to encompass 4 305-d lactations, each one with 60-d dry period. The probabilistic events considered for all scenarios (branches of the tree) were spontaneous embryo reduction, early pregnancy loss, abortion, metritis, retained placenta, and culling rate at 120 d of the second, at the end of the second, and at the end of the third lactation. The partial budget analysis included extra income that was calculated as the aggregation of milk income over feed cost, income from calves born, and slaughter value when culling, whereas extra expenses or losses included additional inseminations, hormonal treatments, embryo reduction, abortion induction, replacement springers, cost due to metritis, and cost due to retained placenta.

**Figure 2:**
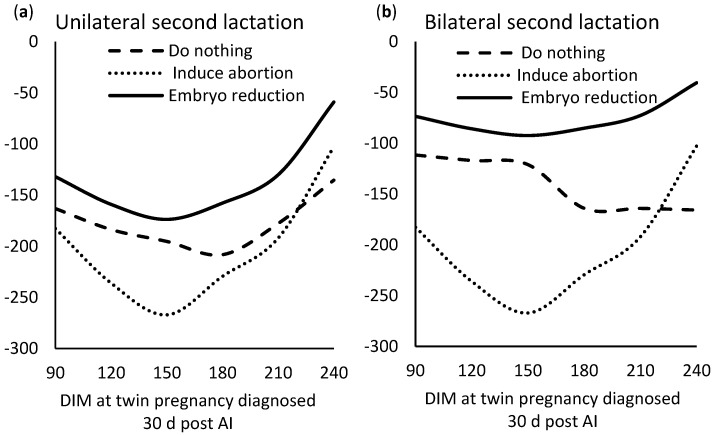
Net cost of a twin pregnancy after three management strategies compared with a singleton pregnancy conceived at the same days after calving (DIM) for unilateral (**a**) and bilateral (**b**) twin pregnancies in second lactation. Source: Adapted from [24].

Ref [24] reported a cost of twinning between $97 and $225 with an average loss value of $161, depending on the type of twin (unilateral or bilateral), parity, and DIM. In general, unilateral twin pregnancies were more costly than bilateral twin pregnancies in early and mid-lactation, whereas unilateral were less costly in late lactation (Figure 2). The negative effect was greater in primiparous cows and during early lactation in bilateral twin pregnancies and during late lactation for unilateral twin pregnancies (Figure 2). The economic loss of twinning was overall greater than in [13] ($112) and [20] ($108). The difference was explained by the fact that [24] accounted for early pregnancy losses, increased culling during current twinning lactation (not only subsequent lactations), finer evaluation at the cow state level (rather than at the overall cow level), and updated milk production levels. [24] estimated that a 1% decrease in twinning would represent between $0.97 and $2.25 profit per pregnant cow and that twinning would have a negative effect of $96 million in the US herd of 9.3 million cows. 

Ref [24] conducted the first study that offers an analysis of possible management options after a twin pregnancy is diagnosed to induce an abortion, reduce an embryo manually, or do-nothing option. The net cost pattern throughout lactation for these three management options was similar in all parities. Figure 2 depicts these patterns for unilateral and bilateral twins for second lactation cows. Induced abortion was consistently the worst economic option during early or mid-lactation, but it was a better option than do-nothing in late lactation when the abortion was close to the insemination cut-off time, as seen in Figure 2. At that time, it increased the culling rate and decreased the risk of extended lactations. On the other hand, consistently, attempting manual embryo reduction was the best economic option for any lactation, any stage of the lactation, and any type of twin pregnancy (unilateral or bilateral, Figure 2) while assuming a success rate of at least 46.2% for unilateral and 71.4% for bilateral twins and even though this management option would increase pregnancy loss by about 20%. During early and mid-lactation, manual embryo reduction decreased the risk of late abortion and all of the negative consequences of twin calving, such as metabolic diseases and decreased lifespan. In late lactation, manual embryo reduction was the best economic alternative, because it decreased the chances of late abortion, eliminated the negative impacts of twin calvings, and decreased the risk of extended lactations. 

Ref [24] also presented breakeven and sensitivity analyses, which were centered in the value of the manual embryo reduction success rate and the potential changes in economic factors, in comparison to do-nothing or inducing an abortion. In short, the breakeven points of success rate for unilateral twins were always below 40% and, for bilateral twins, below 60%, which are well below the reported success rates reported in the literature. Therefore, it was concluded that embryo reduction is a good economic alternative. 

**Figure 3:**
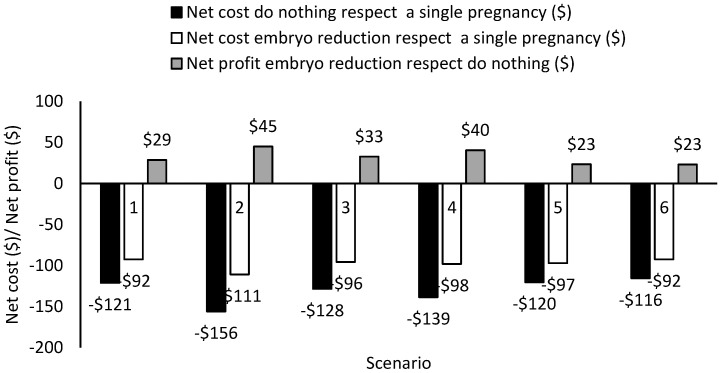
Net cost of different management strategies with respect to a single pregnancy, net profit of embryo reduction to do nothing for different scenarios for cows diagnosed with a bilateral twin pregnancy at 150 DIM. Scenarios: (1) baseline scenario when cow and herd average produce 9979 kg/year, milk price at $0.36/kg, conception rate at first resynchronization (CR) after pregnancy loss 35%, 21-d pregnancy rate after resynchronization (21d PR) after pregnancy loss 25%, days of gestation when twin pregnant cow lose gestation (DG) after do nothing strategy 75 d; (2) cow yielding 454 kg more than the baseline; (3) cow and herd average yielding 454 kg more than the baseline; (4) milk price increased by $0.05/kg milk; (5) CR and 21d PR reduced 5%; and, (6) DG at 52 d. Source: Adapted from [24].

Manual embryo reduction also remained the best option under several plausible and extreme production, performance, and market scenarios evaluated in a sensitivity analysis for bilateral twin pregnancies (Figure 3).When compared to do-nothing, manual embryo reduction always resulted in a positive economic value, even when they chose the most unfavorable situation for manual embryo reduction of a bilateral twin pregnancy at 150 days after calving (DIM). In Figure 3, scenarios 2 (greater cow production), 3 (greater cow and herd production), and 4 (greater milk price) increase the negative economic impact of a twin pregnancy, while it increased the net profit of manual embryo reduction with respect to do-nothing, because the net value of the cow increased and, subsequently, the value of attempting manual embryo reduction, especially among the highest yielding cows in the herd. With a decreased fertility after pregnancy loss (scenario 5, Figure 3), the net profit of manual embryo reduction with respect to do-nothing decreased, because it increased the culling risk. Even in the best possible situation in which the twin pregnancy loss undergoes earlier during gestation (52 d, scenario 6, Figure 3) when the net cost of do-nothing is at its minimum, the manual embryo reduction still remained the most economical. The authors concluded that attempting manual embryo reduction is the most economical option under a wide variety of realistic scenarios.

## 7. Conclusions

Twinning in dairy cows is not desirable because of the negative effects on both cows that calve twins and calves born as twins that result in heavy economic losses. Based on multiple economic analyses that were conducted by numerous researchers, economic losses due to twinning are estimated to range between $59 to $161 per twin pregnancy. Research on twinning that has classified twin pregnancy based on embryo location within the uterine horns reports that economic losses are greater for unilateral than for bilateral twins. Hormonal manipulation before artificial insemination that allows for timed artificial insemination can be a primary strategy for decreasing twinning in high producing Holstein cows before it occurs by decreasing the incidence of double ovulation thereby decreasing the conception of dizygotic twins and associated negative economic consequences of twinning. When twins are diagnosed on farm during early gestation, management options might include doing nothing, terminating the pregnancy, or attempting manual embryo reduction. Based on a recent economic analysis of these options, attempting manual embryo reduction decreased the economic losses of a twin pregnancy by $23 to $45. 

## Figures and Tables

**Table 1:** Partial budgeting of a twin pregnancy in dairy cattle, including the losses, the gains, and the net loss, as reported by different published papers.

Reference	Losses (US$)	Gains (US$)	Net Loss (US$)
[13] ^1^	−212	+100	−112
[20]	−171	+63	−108
^2^ [24]			−161
Unilateral			−200
Bilateral			−150
First lactation			−180
Second lactation			−130
>Second lactation			−120
[26]	−59		−59

^1^ Converted from £ to US$ by multiplying it by 1.33. ^2^ Approximated values from figures.

**Table 2:** Effect of laterality of twin pregnancy on rates of pregnancy loss before Day 90 for control cows (no manipulation) and cows subjected to manual twin reduction followed by progesterone treatment for 21 d (adapted from [25]).

Item	*n*	Loss Rate before 90 d/% (n/n)
Unilateral twin pregnancy	27	
Control	14	64 (9/14)
Manual twin reduction	13	54 (7/13)
Bilateral twin pregnancy	28	
Control	14	0 (0/14)
Manual twin reduction	14	29 (4/14)

## Data Availability

Not applicable for a review study.
